# Early Life Adversity as a Risk Factor for Fibromyalgia in Later Life

**DOI:** 10.1155/2012/140832

**Published:** 2011-10-12

**Authors:** Lucie A. Low, Petra Schweinhardt

**Affiliations:** Alan Edwards Centre for Research on Pain, McGill University, 3640 University Street, Montreal, QC, Canada H3A 2B2

## Abstract

The impact of early life events is increasingly becoming apparent, as studies investigate how early childhood can shape long-term physiology and behaviour. Fibromyalgia (FM), which is characterised by increased pain sensitivity and a number of affective co-morbidities, has an unclear etiology. This paper discusses risk factors from early life that may increase the occurrence or severity of FM in later life: pain experience during neonatal life causes long-lasting changes in nociceptive circuitry and increases pain sensitivity in the older organism; premature birth and related stressor exposure cause lasting changes in stress responsivity; maternal deprivation affects anxiety-like behaviours that may be partially mediated by epigenetic modulation of the genome—all these adult phenotypes are strikingly similar to symptoms displayed by FM sufferers. In addition, childhood trauma and exposure to substances of abuse may cause lasting changes in developing neurotransmitter and endocrine circuits that are linked to anxiety and stress responses.

## 1. Introduction

The causes and underlying pathologies dictating and affecting the development of fibromyalgia (FM) are not yet clear, but as a syndrome affecting between 2 and 4% of the population, with a higher incidence in women [1, 2], it is a hot topic in pain research at this time. As discussed in detail elsewhere in this paper, FM constitutes a chronic pain syndrome, concomitant with a myriad of symptoms, including muscular stiffness and tenderness at specific locations, chronic fatigue, cognitive and mood disturbances, and insomnia. Documented pathophysiologies related to FM include aberrations in neuroendocrine systems, dysfunction in stress regulation and neurotransmitter function, and alterations in brain structure and connectivity. In addition, FM is linked to psychosocial and environmental triggering factors. This paper will explain and discuss some of the potential risk factors of early life adversity (ELA) that display markedly similar outcomes that constitute some of the later symptoms of FM. Pain researchers have clearly shown that noxious events during early life can cause a number of long-lasting changes in pain processing systems in the older organism, which could contribute to the increased pain sensitivity noted in FM patients. In addition, factors such as premature birth and related exposure to stressors, maternal deprivation, and physical or substance abuse in the perinatal period can influence developing neurobiological and psychological states in a number of ways, often causing changes in adulthood similar to the disturbances seen in FM sufferers. Early life pain, hospitalisation, deprivation, emotional trauma, and abuse are discussed in this paper, with speculation about their potential impact upon fibromyalgia.

## 2. Risk Factor: Painful Experiences during Infant Development

It is well established that the experience of pain during infancy causes long-lasting alterations in pain processing that extend well into childhood and adulthood [3–5]. This adds weight to the need for effective understanding and management of pain in neonates, in order to minimise later consequences of early pain experience (see [6, 7]). Infants born with preexisting illnesses or under difficult circumstances, born preterm or needing early surgery, may all need hospitalisation for treatment. In addition, the incidence of premature birth and the necessary associated critical care has increased over the last 20 years [8], and the technology to aid survival has meant that infants as young as 24 weeks postmenstrual age (PMA) can survive and develop on the neonatal intensive care unit (NICU). In these clinical settings, multiple painful procedures may be performed daily for routine monitoring, in addition to any necessary surgeries infants require—for example, Simons et al. [9] found that during the first 14 days of hospitalisation, neonates were subject to an average of 14 painful procedures per day. A more recent study [10] confirmed these findings, showing that neonates were exposed to a daily average of 16 painful and/or stressful procedures and that up to 80% of children were not given specific analgesia for these procedures. This high number of procedures, perhaps repeated over a number of weeks or months, affects the developing nociceptive circuitry of the infant in ways that cause long-lasting changes in pain processing (see [3]) and could explain some of the abnormalities in pain processing displayed by FM patients. 

### 2.1. The Effect of Painful Stimulation on the Human Neonate

Studies of ex-premature children provide compelling evidence for the long-term effects of human early pain experiences. Walker et al. [11] recently presented data on sensory sensitivity in a cohort of 307 extremely preterm infants born at less than 26 weeks postmenstrual age (PMA) in 1995 and followed throughout their lives so far (the UK EPICure cohort). Quantitative sensory testing (QST) established sensory thresholds in these children at age 11 and showed that these extremely preterm children had significantly *decreased* sensitivity to non-noxious mechanical and thermal stimuli compared to age- and sex-matched term born controls. A similar result has been seen in 9–12-year-old children who had previously experienced neonatal cardiac surgery—subjects were significantly less sensitive to nonnoxious mechanical and thermal stimuli at both the previously operated site and noninjured areas [12]. The foundations for this baseline hyposensitivity may be laid whilst children are being cared for on the NICU—following children with NICU experience at 4, 8, and 18 months of (corrected) age, dampened pain responses to immunisation and blunted nociceptive sensitivity to everyday bumps are seen, compared to full-term controls with no NICU experience [13, 14]. Hermann et al. [15] also found elevated heat pain thresholds (i.e., decreased sensitivity) in children who had been hospitalised for a prolonged period as infants and had undergone repeated painful procedures as part of their treatment.

Importantly and more relevant to FM, when pain-exposed neonates are reexposed to noxious stimuli in later life, *hyper*sensitivity to the stimulus is observed. For example, when ex-NICU infants were tested on their perceptual sensitisation to heat pain, where a constant temperature is given for 30 seconds and the change in perception gauged at the end, neonatally hospitalised children showed increased sensitisation compared to nonhospitalised controls, who habituated to the thermal stimulus [15]. Interestingly, heat pain thresholds of all groups of children were increased in the presence of the children's mothers [16], highlighting the importance of social support on the pain experience (which has also been shown to be effective in alleviating the impact of FM [17]). More studies confirm the hypersensitivity seen in ex-premature infants after noxious stimulation in the older child. For example, behavioural sensitivity to noxious mechanical stimuli at the heel persists for at least the first year of life after repeated NICU heel lance experience [18]. Deep somatic and visceral noxious stimulation resulting from early invasive surgery leads to sensitisation of pain responses to later surgery, particularly in regions of the body served by the same spinal nerves as those affected by the initial surgery [19]. These effects are not limited to surgical pain. Children who suffered from burn injuries in infancy (6–18 months of age) showed lower mechanical pain thresholds and greater perceptual sensitisation to both heat and mechanical pain stimuli at sites not originally affected by the burn at ages 9–16 [20]. An interesting study by Buskila and colleagues [21] may be particularly relevant to adult fibromyalgia patients: this study showed that ex-NICU neonates had, as 12–18 year olds, significantly more “tender points” and lower tenderness thresholds than matched full-term children. Seeing as FM diagnosis has been partly based on soreness at a certain number of “tender points”, it could be informative to follow these adolescents over time and assess later FM prevalence. 

Summarizing the above, hypo- as well as hypersensitivity has been observed as a consequence of early life pain. The way in which sensory processing is altered by early life pain may be dependent on several factors. For example, the developmental time point at which injury is experienced can dictate the lasting effects of these injuries (see discussion of the “critical period” in the section “Early Life Exposure to Pain May Influence Fibromyalgia”). In addition, the type of noxious insult (surgical, burn, etc.) and therefore the relative proportion of nociceptors that are activated (i.e., C fibre or A delta nociceptors) may influence the exact nature of altered sensory processing. Finally, as detailed in the following section, it may be that discrete CNS systems are responsible for decreases in tactile and thermal sensitivities and the hyperalgesia seen in neonatally injured humans. 

Animal models of neonatal pain show markedly similar effects as those seen in humans and are crucial to identify and understand cellular mechanisms that are impossible to study in humans. The development of nociceptive circuitry has been studied in-depth, and the neurobiology underlying long-lasting changes is becoming increasingly clear (see [3]).

### 2.2. The Neurobiology Underlying Long-Term Effects Can Be Studied in Animal Models

The utility of animal models is illustrated by evidence showing that the generalised hyposensitivity to mechanical and thermal stimuli shown in humans with early life pain experience (e.g., [14]) is likely mediated by changes in the brainstem regions that modulate ascending afferent input, in particular the periaqueductal grey (PAG) and rostroventral medulla (RVM), which can either enhance or suppress nociceptive input from the spinal cord [22–25]. In the rat, these areas (PAG and RVM) mature over the first three weeks of age [26–28], which corresponds approximately to the time span from the third trimester of gestation to adolescence in human [29, 30]. La Prairie and Murphy [31] injected carrageenan (which causes short-term inflammation lasting around 24 hours) into the hindpaw of male and female rats on the day of birth and saw that, in adulthood, the animals showed decreased sensitivity to thermal stimulation in both the previously injured and uninjured paws. In addition, increased levels of endogenous opioid mRNA were seen in the PAG of the adult animal, and blockade of brain opioid receptors with naloxone abolished this decreased pain sensitivity, leading the authors to speculate that neonatal inflammation induces an upregulation in endogenous opioidergic tone that is maintained into adulthood, so that the adult displays a system that is constitutively “dampening” afferent spinal cord input, leading to decreased pain responses [32]. 

This suggests that early pain can alter endogenous pain inhibitory circuitry. However, FM patients show hyperalgesia rather than hyposensitivity [33–36]. Enhanced nociceptive responsiveness is consistently observed in neonatally injured animals upon adult reinjury and likely results from a number of changes in nociceptive circuitry induced by early pain exposure, all of which result in a nervous system “primed” to respond in an enhanced manner to a new insult. For example, neonatal inflammation causes thermal hyperalgesia in rat pups that lasts from several weeks up to adulthood [37–39]. This inflammation and concomitant release of inflammatory and trophic molecules results in enhanced spinal neuronal responses to paw pinch in the adult, as well as increased primary afferent nerve fibre innervation of the dorsal horn of the spinal cord [40]. Inflammation-induced alterations in the developmental connectivity of the spinal cord [41] and/or sprouting of nerve fibres at the skin also result in an increased nociceptive response [42]. Neonatal skin wounds, like inflammation, cause drops in mechanical withdrawal thresholds at the site of injury and increase in dorsal horn receptive field size weeks after the wound had healed [43, 44], as well as causing release of nerve growth factors leading to hyperinnervation of the skin, and increased sensitivity to noxious stimuli in later life [45]. This is consistent with human studies showing hypersensitivity after injury in children, especially those with previous surgical history [19, 46, 47]. Therefore, the apparent contradiction between generalised decreased sensitivities and hyperalgesic responses to new noxious stimulation may in part be due to discrete CNS processing systems dictating behavioural responses, for example, enhanced endogenous pain inhibition at a brainstem level, but increased hypersensitivity/hyperinnervation at a spinal level.

### 2.3. Early Life Exposure to Pain May Influence Fibromyalgia

As explained above, whilst La Prairie and Murphy [31] showed that animals subject to neonatal inflammation showed generalised hypoalgesia to thermal stimuli at baseline in adulthood, when animals were reinjured as adults, animals were *more *sensitive to noxious thermal stimulation. Importantly, all of these effects were greatest in female animals. This finding might be relevant to fibromyalgia syndrome, as the incidence of FM is greater in women [48]. Furthermore, disturbances in descending pain modulation have been reported in FM patients [49, 50] and patients show decreased blood serum levels of serotonin and lower CSF levels of serotonin and noradrenaline metabolites [51–54]. This may be relevant to FM, as there is a discrete descending serotonergic system projecting from the RVM that modulates spinal cord excitability [55–59]. Furthermore, noradrenergic signalling, originating from the locus coeruleus, has a role centrally in feedback inhibition of pain (see [60]). 

The developmental timing of any injury determines potential long-term effects, leading to the concept of a “critical period” of nociceptive development, within which pain experience permanently alters pain processing (see [5, 61]). To illustrate, giving a skin incision to the hindpaw of a neonatal rat at postnatal days (P) 3 or 6 produces an increased pain response to a repeat incision 2 weeks later. If, however, the initial incision is performed after the critical period (at P10, 21, or 40 followed by repeat incisions 2 weeks later), the enhanced hypersensitivity to the later incision is not seen [61]. Understanding the concept of a “critical period” of nociceptive development may be useful for determining some of the root causes of fibromyalgia—as human infants born prematurely display long-term alterations in pain processing, it is possible that early pain experience within this time window contributes to some of the adult pain in FM. At this time, there is very little literature that attempts to delve into the neonatal and childhood life of current FM patients.

### 2.4. Adequate Pain Management of Neonates May Decrease FM Prevalence in Adults

In order to prevent these physiological disturbances, adequate pain management for human neonates is an important clinical issue. Pain management of neonates is a difficult area, as neonates cannot give verbal feedback on their pain experience, and are physiologically very different to the adult state that dictates dosage, metabolism and efficacy (i.e., [7]). Current treatments include morphine and benzodiazepines both for postsurgical pain and general sedation on the NICU [62], as well as nonpharmacological interventions such as the administration of sucrose for acute procedures including heel lance [63] (although the effectiveness of these interventions and the long-term effects of chronic exposure to sucrose and drugs such as morphine are not yet clear [64, 65]). The effects of these continue to be studied, and given the long-term effects of early pain as discussed above, adequate pain management for neonates might reduce various pain syndromes in later life, including fibromyalgia. The importance of adequate analgesia for neonatal procedures is illustrated in a seminal paper by Taddio et al. [46]. They performed a double-blind, randomized, controlled trial (RCT) on the effects of a topical anaesthetic (EMLA cream) used during male neonatal circumcision, which has traditionally been done without anaesthesia or analgesia. Looking at pain responses in the infants when they were later vaccinated at 4–6 months, boys who had been treated with the EMLA cream when circumcised showed lower pain responses than those who received no anaesthesia, and the circumcised groups both showed higher pain scores than uncircumcised controls.

## 3. Risk Factor: Premature Birth and Related Stressors

As discussed above, pain during the neonatal period can alter the nociceptive processing pathways of an organism for life, potentially impacting upon the development of fibromyalgia in later life. Many of the human studies mentioned in the previous section recruited infants born prematurely, needing the intensive care unit for survival. The NICU is a strange and abnormal environment in comparison to the womb, and premature infants are exposed to many stressful stimuli in addition to repeated nociceptive procedures, such as light, noise, tactile stimulation, surgery, medication, and maternal separation, all of which could feasibly affect development [66]. Indeed, even the act of nursing very premature infants (changing diapers etc.) causes increases in stress hormones [67]. In addition to the long-term effects of increased pain sensitivity in these children are effects upon stress regulatory systems, where a large body of evidence suggests that prematurity and the resulting experiences on the NICU can permanently alter in particular the hypothalamic-pituitary-adrenal (HPA) axis. As this is shown to be disturbed in FM patients [68–71], it is possible to speculate that premature birth in itself may influence the occurrence of FM in adults. 

### 3.1. Premature Birth Impacts upon the Body's Response to Stressors

The HPA axis is the body's stress-response system. Cells of the hypothalamus produce corticotropin-releasing factor (CRF) in response to an environmental stressor, and a cascade of events ultimately causes adrenaline release and the production of the “stress hormone” cortisol. Under normal circumstances, adrenaline and cortisol release is terminated via a negative feedback circuit. In FM, however, HPA axis regulation appears to be abnormal. Whilst the precise dysfunctions in stress regulation via the HPA axis are not clear at this point (some studies find FM patients show hypocortisolism (see [72]), whilst others describe hypercortisolism and HPA hyperactivity [68, 69, 73]), what is clear is that HPA axis function is not normal in many patients. The discrepancy between findings of hyper- and hypocortisolism may be due to a number of factors. For example, disease-specific patient characteristics, such as symptom profiles and comorbidities, as well as disease-nonspecific characteristics, such as age, gender, personality traits, and socioeconomic background, can affect results. In addition, markers of HPA axis function differ between studies (e.g., basal levels or evoked cortisol responses) as do the time points of measurement (e.g., upon waking or following diurnal fluctuations), in addition to other technical details. In instances where the literature does not permit any conclusions on the direction, we refer to *alterations* in HPA axis function, rather than increases or decreases.

Nevertheless, the way in which premature birth alters cortisol levels and cortisol responses compared to term-born controls is relatively unambiguous. Grunau et al. [66] propose that early stressors such as those routinely experienced in the NICU can impact upon development of the HPA axis by causing consistent release of adrenaline and cortisol and increase the “allostatic load” of the neonate—the concept of “allostasis” explaining how an organism's physiological systems fluctuate over time in order to meet the demands of external stressors, in an attempt to regain homeostasis (bodily equilibrium). The impact of chronic stress and the accompanying neuroendocrine responses may also ultimately cause long-lasting damage to bodily organs and contribute to chronic disease development [74]. In preterm neonates, this may manifest as a life-long shift in HPA axis balance. 

Basal cortisol levels are often low in neonates on the NICU in comparison to term infants, which is unexpected considering the length of time that infants spend there and the stressful procedures the still-developing neonate is subject to [75, 76]. Grunau and colleagues have published a series of studies giving convincing evidence that NICU experience causes “resetting” of the endocrine stress systems, by measuring cortisol levels after noxious experiences in ex-preterm infants, either whilst still on the NICU (short-term effects), or in the months to years following. When a clinically required heel lance was done whilst infants were still on the NICU, the earliest premature infants born at less than 28 weeks gestational age (i.e., approximately 3 months premature) showed a dampened cortisol response to heel lance. In addition, higher cumulative exposure to neonatal procedural pain over the length of stay in the NICU was related to lower cortisol release to standard nursing procedures [77]. When immunised at 2–4 months (corrected) age, low gestation age (LGA) boys (<32 weeks), but not girls, showed lower cortisol concentrations than full-term infants after injections, although facial and heart rate responses did not differ between groups [78]. 

When studied over a longer period of time, we see that this early dampened cortisol response in premature infants changes to elevations in cortisol levels and responses when the children are older. At 8 months old, infants born at extremely low gestational age (≤28 weeks) with previously low basal cortisol levels, showed elevated basal levels as well as greater increases in cortisol response to stressors, compared to term infants. In these children, greater increases in cortisol were associated with higher numbers of skin-breaking procedures experienced in the past on the NICU [79]. Grunau et al. [80] followed the time course of this “switch” from low to high stress hormone levels and found that at 3 months corrected age, basal cortisol levels were lower than term controls, but, at 8 and 18 months, the youngest ex-premature infants had significantly higher cortisol levels than term controls. The authors speculate that the HPA axis has been “reprogrammed” by NICU experience. Recent work has replicated the finding that premature children born onto the NICU later have higher basal cortisol levels compared to term-born controls at both 18 months [81] and upon waking in 8–14 year old ex-premature infants [82]. 

Animal studies support the existence of a developmental shift from low to high cortisol levels after perinatal corticosteroid exposure, and these higher levels of cortisol seen in older animals are associated with increased levels of corticotropin-releasing hormone (CRH) mRNA and glucocorticoid (GC) receptors in the amygdala (e.g., [83, 84]). In humans, this shift from low to high levels over development may be influenced by the fact that the mothers at risk of giving birth prematurely are routinely given corticosteroids to delay birth and enhance infant survival and lung function—an intervention which suppresses cortisol secretion in the infant when born [85] yet causes an increased cortisol response after heel lance at 24 hours after birth [86]. Further work is needed to address the impact of perinatal glucocorticoid exposure on the stress response axis in later life.

### 3.2. Prematurity May Contribute to Adult FM Symptoms via the HPA Axis

The above evidence highlights how premature birth and the stressors associated with it can influence the physiological response to stress and “reset” the balance of the HPA axis response. Seeing as FM patients routinely show imbalances in the stress response, it is reasonable to hypothesise that premature birth may be a risk factor for developing FM in later life. Indeed, Klingmann et al. [87] show that of 93 female FM patients, 62% reported a gestation length of <38 weeks, which was related to a lower cortisol response upon waking when compared to full-term FM patients. The authors speculate that enhanced glucocorticoid levels in the mother during pregnancy or in response to premature birth affect the development of the adrenal glands in the foetus/premature infant, rendering the HPA axis less capable of dampening stress responses to later stressors. This in turn may disinhibit responses to physical or psychological stress and affect brain function, resulting in enhanced responses to pain and increased fatigue levels. Support for this hypothesis comes from animal studies of prenatal glucocorticoid exposure, where dam rats are exposed to substances that increase HPA axis activity (such as glucocorticoid receptor agonists), in the third trimester of gestation. Results show that the adrenal glands and brain weight of the adult offspring are smaller, stress regulation is compromised, and cognitive dysfunction is seen in tests of memory as well as anxiety-like behaviour, with the effects exacerbated in female offspring [88–93].

### 3.3. Cognitive Symptoms of FM May Arise from Differences in Brain Development Caused by Premature Birth

One symptom of FM, colloquially called “fibro-fog” by sufferers, constitutes cognitive deficits, with patients complaining of difficulties in memory and attention that mimic the effects of an extra 20 years of ageing (e.g., [94, 95]). Additional evidence that prematurity may influence the development of FM comes from studies showing that the risks of cognitive and psychiatric impairment are much greater in ex-preterm infants. The EPICure cohorts (born at ≤25 weeks gestation) have recently had their cognitive abilities and psychiatric profiles investigated at 11 years of age. Results showed that the children born at the youngest preterm ages are at higher risk of ADHD, autism spectrum, and emotional disorders [96] and show increased incidences of learning impairments and poor academic attainment [97]. Other meta-analyses and epidemiological studies have confirmed the increased risk for psychiatric symptoms and poorer academic performance in older childhood after premature birth [98–100]. 

Brain imaging of ex-preterm infants compared to full-term controls has shown underlying changes in brain structure and function that may help explain some of these deficits. Cortical surface area is decreased at full-term in extremely preterm infants [101], and the incidence of white matter abnormalities persisting past 18 months (corrected) age is increased [102]. Thalamic volume is also reduced in preterm children at term-equivalent age [103] and at 2 years of age, and connectivity between the thalamus and cortex may be disrupted in ex-preterm children [104]. Seeing as the premature brain is still developing at a rapid pace and as myelination occurs during late preterm maturation [105], it is likely that prematurity influences white matter development, helping to explain why later cognitive deficits may arise. If premature birth becomes a proven risk factor for fibromyalgia, the neural bases of “fibro-fog” may become better understood in the adult.

## 4. Risk Factor: Maternal Deprivation

As discussed above, early pain experience and prematurity may be risk factors for FM development in later life. Prematurity might be a risk factor in combination with the high exposure to additional stressors, including maternal deprivation. Relatively detailed information is available on the effects of maternal deprivation on the developing organism, and therefore maternal deprivation is discussed separately. Animal models of deprivation have proven extremely useful in illustrating the effects of deprivation from the primary caregiver (generally the mother) and the strong role for the fluidity of genetic expression during early life in the shaping of the adult phenotype. The study of epigenetics, or how the environment influences the activation and expression of different genes, has provided fascinating insights into this fluidity.

### 4.1. Maternal Deprivation in Animal Models Influences Later Stress Responses

Animal models of maternal deprivation often use rats and generally employ a paradigm whereby pups are separated from the mother for at least an hour per day, much longer than the 20–25 minutes of absence that dam (mother) rats are routinely away from the nest [106]. When neonatal rats are exposed to these prolonged periods of deprivation during the first weeks of life, a number of physiological and behavioural changes occur in the adult animal. For example, rats separated from the dam for 180 minutes per day from postnatal day (P) 2–14 show elevated levels of CRF mRNA as adults, which causes adrenaline and cortisol release via activation of the HPA axis [107, 108]. They also show more anxiety-like behaviours as adults and an increased propensity to consume alcohol [109, 110]. Accordingly, maternal deprivation has now been used to model various psychiatric states such as anxiety [111], addictive disorders [112], and schizophrenia [113], and a recent study by Uhelski and Fuchs [114] showed that maternally deprived animals showed increased active avoidance of environments in which pain had been experienced, suggesting enhanced supraspinally mediated responses to pain. The importance of maternal presence is further illustrated by work with monkeys: infants reared in the absence of an adult caregiver but together with age-matched peers develop chronic anxiety-like behaviours and disordered cortisol levels to stressors (suggesting HPA axis imbalance), similar to FM symptoms [115]. Taken together, these data show that maternal deprivation has been associated with three important disturbances found among FM patients, namely, alterations in the HPA axis, increased anxiety, and increased pain responses [116]. 

Short-term separation of pups conversely causes opposite effects to longer maternal deprivation. If pups are subject to only 15 minutes of deprivation, the adult animals display more social contact and better stress-coping abilities compared to animals deprived for longer periods [117–121]. These effects seem to be mediated by the maternal style of the dam upon reunion with the pups—mothers of pups separated for short periods engage in more licking/grooming and arched-back nursing (LG-ABN) when reunited compared to dams of pups separated for more prolonged periods. Dams that engage in high levels of this nursing style produce offspring that show more efficient stress regulation, as measured by corticosterone (the animal equivalent of CRH) responses to stress and feedback sensitivity of the HPA axis [122, 123]. In fact, the maternal LG-ABN style (either high or low) causes individual differences in stress responsiveness and emotionality that remain stable in the adult offspring [124] and is in itself a trait that is passed on to female offspring. Cross-fostering studies, where pups from low or high LG-ABN dams are reared by dams showing the opposite LG-ABN behaviour illustrate that female offspring will show nursing styles akin to their “foster” dam rather than their biological dam [125]. Further evidence for the fluidity of these behavioural phenotypes comes from evidence that low LG-ABM dams rearing litters in a socially-enriched environment produce offspring showing enhanced exploration and licking/grooming behaviour of their own offspring [126]. 

The effects upon adult phenotype that depend on maternal style are regulated by changes in DNA methylation of the infant genome, leading to activation or silencing of certain genes, and alterations in levels of, for example, glucocorticoid receptors [122], neurotrophic factors and specific neurotransmitter receptors in the hippocampus [127]. Serotonin (5-HT) turnover (as seen by measures of 5-HT levels compared to levels of 5-HT metabolites) is also increased in maternally deprived animals [128], and expression and levels of serotonin receptors and transporter proteins altered [129, 130]. This is particularly relevant to FM, as the serotonergic system has been implicated in the affective components associated with FM—cerebrospinal fluid levels of 5-HT metabolites are decreased in patients [53], serotonin antagonists are effective drugs for some FM patients with no associated depressive comorbidities [131], and increased incidence of a specific genetic polymorphism in the 5-HT transporter gene has been identified in FM patients [132], although the exact disorders in serotonergic signalling are not yet clear [133]. However, some of the heterogeneity in FM patients may arise due to epigenetic alterations that occurred during early infancy. To date, no research has been conducted on this specific question.

### 4.2. Quality of Maternal Attachment Affects Pain Processing

The quality of the relationship between child and primary caregiver (generally the mother) can also dictate emotional reactivity throughout life and the type of attachment style that an individual will form with others throughout their life. Bowlby first developed the idea of “attachment theory”, studying the bond between child and mother and suggested that a secure attachment style is the most beneficial for infant development [134, 135]. Since then, studies have shown that disordered attachment styles are linked to chronic pain and problems coping with pain (see [136]). For example, chronic pain patients with high levels of avoidant attachment self-scored pain intensity more highly, and patients with fearful attachment styles display increased levels of pain catastrophising, linked to anxiety levels [137, 138]. In acute pain tests, adults showing secure attachment styles rated pain as less intense and anxiogenic [139]. Importantly for FM, secure attachment formation is linked to the dopamine and opioidergic system in both animals and humans [140, 141], suggesting that a secure early attachment between infant and parent could be protective against developing FM in later life. Hallberg and Carlsson [142] indeed mention the overrepresentation of individuals with insecure attachment styles in the chronic pain patient population.

## 5. Risk Factor: Childhood Physical and Psychological Trauma

Physical and sexual abuse during childhood are well-documented risk factors in the development of fibromyalgia [143–146], and two recent meta-analyses link childhood incidence of physical and sexual abuse with FM [147, 148]. Early life abuse carries with it the burden of a number of other behavioural and pathological problems, including increased incidence of depression, posttraumatic stress disorder, alcoholism, substance abuse, obesity, ill health, and suicide ([149; see 150]). A number of these are also comorbidities in FM. It is possible that the impact of early abuse and trauma contributes to FM via disruption of neurotransmitter systems such as the serotonergic and dopaminergic systems and impacts stress-management via the HPA axis [151–155]. 

### 5.1. A High Incidence of Childhood Abuse Is Reported in FM Patients

Self-report studies, where patients are questioned on their childhood history, consistently show increased early life adversity in FM patients. Goldberg et al. [156] found that three different groups of chronic pain patients (facial pain, myofascial pain, and FM), all had a history of abuse in nearly 50% of cases, rising to 65% in the fibromyalgia group. In particular, females with an alcoholic parent were likely to be members of the FM group. Hallberg and Carlsson [142] conducted in-depth interviews with 22 FM patients and describe “abundant examples of early loss (and...) high degree of responsibility early in life,” and Anderberg et al. [157] found that, of 40 female FM patients, 51% had experienced very negative childhood or adolescence life events, compared to 28% in healthy age-matched women. Nicolson et al. [158] found an association of self-reported childhood abuse and neglect with FM patients' daily cortisol levels, finding the most disordered cortisol responses in the patients reporting the highest levels of sexual and emotional abuse. This suggests that early abuse further impacts upon the HPA axis, which shows aberrant functionality in FM in patients with no history of abuse [71]. Childhood rape has also been strongly associated with a lifetime diagnosis of FM [147]. 

The loss of a parent during early childhood is an emotionally traumatic event and is associated with altered daily cortisol levels in the adult, particularly in men [159, 160]. Poor quality family relationships during childhood also cause changes in cortisol release in response to a stressful event [161]. In addition, early-life stress and trauma has been shown to be a significant predictor of levels of CRF in cerebrospinal fluid (CSF) in non-FM subjects [162], and Danese et al. [163] found an association between early-life maltreatment and adult blood levels of C-reactive protein, a marker of inflammation. Specific to fibromyalgia, McLean et al. [164] showed that women with FM who reported sexual or physical abuse in their personal histories had differences in CSF levels of CRF compared to nonabused FM women, and Weissbecker and colleagues [165] found that a similar group of FM women abused in childhood had disordered diurnal cortisol levels. 

However, studies based on the self-report of FM patients can be difficult to verify. Adult life experiences may bias memories from early life, and it is possible that FM patients, due to their current pain and comorbid states, become self-centred and preoccupied with the pain, and potentially overemphasise traumatic memories [166, 167]. Longitudinal studies following children and young people who have suffered from documented abuse, for example, children in social care, would be useful in order to avoid this confound. Epidemiological studies of socioeconomic position (SEP) during childhood have shown that lower SEP during childhood is a predictor of chronic widespread pain in later life [168]. Whilst socioeconomic status incorporates a large number of factors, trauma-related hospitalisations are more prevalent in children from lower socioeconomic statuses who have less access to appropriate healthcare [169–172].

## 6. Risk Factor: Perinatal Exposure to Substances of Abuse

Serotonin is not the only neurotransmitter system altered in fibromyalgia—dopamine and opioid neurotransmitter disturbances are also reported that may, in part, result from interference with these developing systems during pre- or early postnatal life. Dopamine responses of FM patients to painful stimuli are lower than those of healthy subjects [173], and low CSF levels of dopamine (as well as serotonin and norepinephrine) metabolites are seen in patients [52]. Furthermore, the opioid system is closely linked to dopaminergic signalling, constitutes an important endogenous antinociceptive system, and is altered in FM [174, 175]. Exposure to a number of substances during early life will impact upon the development of these neurotransmitter systems. For example, exposure of the foetus to alcohol induces dysfunction in dopaminergic and serotonergic systems and will persistently affect the development of the HPA axis [176–178], all of which are, as mentioned, disturbed in FM. 

### 6.1. Early Opioid Exposure Causes Long-Lasting Changes in Nociceptive Systems

Early exposure to opioids, such as exposure of the foetus to heroin or methadone during pregnancy, or prolonged morphine administration after birth, for example, on the NICU, causes lasting changes in opioid signalling. If exposure occurs prenatally, it can result in the newly born infant undergoing withdrawal symptoms very soon after birth, the so-called neonatal abstinence syndrome (NAS) [179]—an effect mirrored in animal models of early drug dependence [180]. Morphine is used in the NICU to provide sedation in neonates requiring mechanical ventilation and to improve tolerance of ventilation and comfort of the infant. The lasting effects of this are not yet well-characterised [65, 181], although it is known that chronic morphine administration causes changes in mu-opioid receptor density and sensitivity that desensitises older animals to opiate analgesia [182, 183]. As the opioidergic and dopaminergic systems are crucial for endogenous modulation of pain, and this modulation seems to be disordered in fibromyalgia, longitudinal studies of early life exposure to opioids may help explain some of the disturbances in opioid function seen in FM. Positron emission tomography (PET) studies would be useful to investigate if the altered opioid receptor activity seen in FM patients is related to early life opioid exposure. Harris and colleagues [175, 184] have shown that FM patients show alterations in mu-opioid receptor availability, but a history of opioid use was one of the exclusion criterion for these studies, meaning that the impact of early life exposure to opioids is not yet known in terms of later FM prevalence.

### 6.2. Dopamine Overexposure during Early Life Affects HPA Axis Function, and May Be Mediated by Epigenetic Factors

Dopaminergic drugs such as cocaine and amphetamine increase anxiety-like behaviours in animal models of abuse during pregnancy [185], and cocaine activates the HPA axis by potentiating adrenocorticotropin hormone (ACTH) release, which in turn stimulates the adrenal glands to produce adrenaline and cortisol [186]. Increasing evidence now suggests that prenatal exposure to drugs of abuse (including alcohol, cocaine, and amphetamine) may be toxic to developing dopamine-rich areas such as the basal ganglia (see [187]). After birth, studies on the development of the dopamine system suggest that functional connectivity in the young animal, particularly to frontal cortical areas, is not mature until adolescence or later [188, 189], and it is possible that the balance between tonic and phasic dopamine release will also be affected by exposure of the developing system to excessive dopamine activation, impacting upon later pain control mechanisms [190]. Therefore, early life exposure to increased levels of dopamine could help explain the aberrant dopaminergic functionality seen in FM patients [173, 191]. Interestingly, genetic polymorphisms linked to FM include the *val^158^met *polymorphism (in the gene coding for catechol-o-methyltranferase, an enzyme that metabolises dopamine) [192] and the dopamine D4 receptor [193]. Considering that epigenetic effects impact upon stress regulation in maternally deprived animals, it is possible to speculate that early life events cause epigenetic changes, which may interact with the above polymorphisms to produce an adult phenotype at increased risk of developing FM. Indeed, self-report studies of FM patients show higher levels of reported parental drug and alcohol abuse in comparison to other groups of other chronic pain patients [194, 195].

## 7. Conclusions

Fibromyalgia is classified as a disorder of pain processing and stress regulation, often along with comorbidities of anxiety and depression; the amount of evidence that links early life adversity to pain, stress, and emotional problems in later life strongly suggests that these adverse events and traumas could increase the risk of developing FM in adulthood. At this point, research has shown that FM patients self-report high levels of early adversity and indicates that early life circumstances affecting later pain processing and stress regulation may be more prevalent in FM. However, there is little evidence to conclusively link the two, for example, by proving that maternal deprivation or increased stress during pregnancy increases the incidence of FM. 

This paper has focused on the evidence showing that painful procedures during early life cause long-lasting changes in pain processing and suggests that high exposure to painful experiences in early life may partially explain the increased pain sensitivity shown by FM patients. In addition, childhood adversities and maternal deprivation are also discussed in terms of the effects they have on stress-regulatory systems in the older organism, so again are potentially sources of the disorders in cortisol levels and stress response seen in FM patients. As previously mentioned, the precise dysfunctions in stress regulation via the HPA axis are not yet clear in FM patients, but what is clear is that the body's stress regulatory systems are compromised, and it is possible that the precise nature and combination of each individual's childhood experience may be contributing to the overall symptomatology of FM. 

Exposure of the developing brain to perinatal stress and glucocorticoids during critical periods of development may affect the long-term function of areas involved in stress regulation such as the hippocampus and amygdala and help explain the “fibrofog” and anxiety disorders prevalent in FM. Furthermore, impairments in stress regulation caused by early exposure to stressors such as increased maternal cortisol levels, pain, or maternal deprivation may also partially explain the increased pain sensitivities seen in FM patients. Finally, as pain is itself a stressor, the pain experienced by FM patients may be acting in a positive feedback manner to further increase anxiety levels and impact upon stress regulation. Whilst FM is unlikely to be due to a single factor, it is possible that the factors outlined in this paper, concomitant with a number of other factors such as a genetic predisposition to enhanced pain sensitivity, stressful life experiences in adult life, and the influence of sex hormones, may combine or interact to create a phenotype at higher risk of developing this form of chronic pain. Teasing apart potential influences and their mechanisms may help treat sufferers or, in fact, decrease the risk of future suffering.
